# Association of body mass index and osteoarthritis with healthcare expenditures and utilization

**DOI:** 10.1002/osp4.398

**Published:** 2020-01-13

**Authors:** Stephen S. Johnston, Eric Ammann, Robin Scamuffa, Jonathan Samuels, Andrew Stokes, Elliott Fegelman, Chia‐Wen Hsiao

**Affiliations:** ^1^ Department of Epidemiology, Medical Devices Johnson & Johnson New Brunswick New Jersey; ^2^ Pre‐clinical, Clinical, and Medical Affairs Ethicon Somerville New Jersey; ^3^ Division of Rheumatology, Department of Medicine NYU Langone Medical Center New York; ^4^ Department of Global Health Boston University, School of Public Health Boston Massachusetts

**Keywords:** body mass index, healthcare expenditures, healthcare utilization, osteoarthritis

## Abstract

**Objective:**

Osteoarthritis is highly prevalent and, on aggregate, is one of the largest contributors to US spending on hospital‐based health care. This study sought to examine body mass index (BMI)–related variation in the association of osteoarthritis with healthcare utilization and expenditures.

**Methods:**

This is a retrospective study using administrative insurance claims linked to electronic health records. Study patients were aged ≥ 18 years with ≥1 BMI measurement recorded in 2014, with the first (*index*) BMI ≥ 25 kg m^−2^. Study outcomes and covariates were measured during a 1‐year evaluation period spanning 6 months before and after index. Multivariable regression analyses examined the association of BMI with osteoarthritis prevalence, and the combined associations of osteoarthritis and BMI with osteoarthritis‐related medication utilization, all‐cause hospitalization, and healthcare expenditures.

**Results:**

A total of 256 459 patients (median age = 56 y) met study eligibility criteria; 14.8% (38 050) had osteoarthritis. In multivariable analyses, the adjusted prevalence of osteoarthritis increased with increasing BMI (12.7% in patients who were overweight [25.0‐29.9 kg m^−2^] to 21.9% in patients with class III obesity [BMI ≥ 40 kg m^−2^], *P* < .001). Among patients with osteoarthritis, increasing BMI (from overweight to class III obesity) was associated with increased (all *P* < .01): utilization rates for analgesic medications (41.5‐53.5%); rates of all‐cause hospitalization (26.3%‐32.0%); and total healthcare expenditures ($18 204‐$23 372).

**Conclusion:**

The prevalence and economic burden of osteoarthritis grow with increasing BMI; primary prevention of weight‐related osteoarthritis and secondary weight management may help to alleviate this burden.

## 1 INTRODUCTION

Osteoarthritis affects over 30 million individuals in the United States and imposes a substantial healthcare utilization and expenditure burden on the US healthcare system.^1^, ^2^ In 2013, osteoarthritis was the second most expensive condition treated in US hospitals, resulting in aggregate hospital costs of over $16 billion, stemming from over 1 million hospital stays.^3^ Osteoarthritis has also been associated with substantial incremental increases in other forms of medical healthcare utilization, need for joint arthroplasty, and use of pain medications including opioid analgesics.^2^, ^4^, ^5^, ^6^

The association between obesity and the incidence and prevalence of osteoarthritis has been well established.^7^, ^8^ Prior studies in which healthcare utilization and expenditures were compared between patients with vs without osteoarthritis have not accounted for the potential role of obesity in such comparisons.^4^, ^5^, ^6^ Thus, there is currently little published information regarding whether, and the extent to which, body mass index (BMI) varies the level of healthcare utilization and expenditures of patients with osteoarthritis in absolute terms and in relation to those without osteoarthritis.

Understanding the role of BMI in this relationship would help to quantify whether opportunity exists to reduce the healthcare burden associated with osteoarthritis, through means such as primary prevention of weight‐related osteoarthritis and secondary weight management. The overall goal of this study was to examine BMI‐related variation in the association of osteoarthritis with healthcare utilization and expenditures.

## 2 MATERIALS AND METHODS

### 2.1 Data source

This was a retrospective, observational, cross‐sectional study. The study data were extracted from the de‐identified Optum Integrated Claims‐Clinical Database (Optum Integrated), which includes administrative insurance claims data linked to electronic health record (EHR) data for a geographically diverse subset of individuals (5.5 million) with commercial or Medicare Advantage health plans provided by a large US health insurance carrier. The insurance claims files include facility, physician, and pharmacy claims submitted for reimbursement on behalf of covered health plan members. The EHR data include records of clinical diagnoses, procedures, body measurements, vital signs, laboratory results, prescriptions, and notes recorded as part of routine clinical practice in EHR systems that contribute data to the Humedica healthcare analytics platform.

### 2.2 Patient selection

Patients selected for study met all of the following criteria: ≥1 BMI measurement recorded in the EHR database from 1 January 2014 through 31 December 2014 (first observed BMI measurement designated *index BMI*); continuous health plan enrolment, as measured from the insurance claims database, for 6 months before and after the index BMI measurement (1‐y *evaluation period*); measured index BMI equalling 25 to 80 kg m^−2^; no missing data for demographic covariates; age ≥ 18 years as of index BMI; and total medical and prescription payments during evaluation period ≥ $0.

### 2.3 Patient classification by BMI and osteoarthritis

Patients were classified into World Health Organization BMI categories on the basis of the index BMI measurement: overweight (BMI 25.0‐29.9 kg m^−2^); class I obesity (BMI 30.0‐34.9 kg m^−2^); class II obesity (BMI 35.0‐39.9 kg m^−2^); and class III obesity (BMI 40+ kg m^−2^).^9^ Patients were classified as having (diagnosed) osteoarthritis, if they had ≥1 medical claim with an *International Classification of Diseases, Ninth Revision, Clinical Modification* (*ICD‐9‐CM*) diagnosis for osteoarthrosis and allied disorders (715.xx) during the 1‐year evaluation period.^10^

### 2.4 Measurement of patient characteristics

Patient demographic characteristics included age, sex, geographic region, and insurance type (commercial vs Medicare), measured as of the index BMI. The Quan et al^11^ adaptation of the Charlson co‐morbidity index was used to measure the presence of selected co‐morbidities during 1‐year evaluation period; this measure was included for descriptive purposes only and was not adjusted for within the multivariable models because of its potential for endogeneity, as described below.^11^

### 2.5 Measurement of healthcare utilization and expenditures

Healthcare utilization and expenditures were measured throughout the 1‐year evaluation period. Healthcare utilization outcomes included the following dichotomous flags for hospitalizations: ≥1 all‐cause hospitalization; ≥1 hospitalization with a primary diagnosis of osteoarthritis; ≥1 hospitalization with a lower‐extremity orthopaedic surgery diagnosis‐related group (DRG) (primary or revision); and ≥1 hospitalization with both a primary diagnosis of osteoarthritis and a lower‐extremity orthopaedic surgery DRG (primary or revision). Additionally, individual dichotomous flags were created for ≥2 prescription fills or medical claims for each of the following osteoarthritis‐related medication classes^12^: opioid analgesics, nonsteroidal anti‐inflammatory drugs (NSAIDs, including selective COX‐2 inhibitors), intra‐articular injections, muscle relaxants, antidepressants, anticonvulsants, anxiolytic/sedative/hypnotic, and a composite category of any centrally acting analgesic medication comprising opioid analgesics, NSAIDS, acetaminophen, and salicylates. The criterion of ≥2 prescription fills/medical claims was chosen to (conservatively) ignore instances in which a given medication class was filled only one time, which may represent only brief or acute use. Medication classes were defined according to the Multum Lexicon taxonomy.^13^

To protect the confidentiality of the payment data of the national insurer from which the insurance claims data are collected, healthcare expenditures in the study database are based on a standardized pricing algorithm which results in an estimate of the allowed amount (insurance plus patient out‐of‐pocket) for a given healthcare encounter (eg, outpatient service or hospitalization) or product (eg, prescription). This algorithm implicitly accounts for healthcare cost inflation over time and reflected 2015 constant dollars at the time that this study was conducted.

### 2.6 Statistical analyses

Bivariate statistical analyses were conducted for all study variables, stratified by BMI category and by presence vs absence of osteoarthritis (where applicable). Multivariable logistic regressions were used to examine the association between BMI category and the prevalence of osteoarthritis, adjusting for patient age, sex, and geographic region. Separate models were fit for each anatomical site of osteoarthritis, according to *ICD‐9‐CM* descriptions: any osteoarthritis site; lower extremity; upper extremity; and site unspecified. Because increasing BMI is presumably associated with an increase in the probability of having other co‐morbidities, as well as an increase in the probability of being insured by Medicare because of disability, these variables were considered to be endogenous and therefore were not adjusted for within the multivariable models.

Separate multivariable logistic regressions were also used to examine the association between BMI categories, osteoarthritis, and the following outcomes measured during the evaluation period: all‐cause hospitalization and each of the osteoarthritis‐related medication outcomes. In these regressions, BMI, osteoarthritis, and an interaction term between these two factors were included, along with patient age, sex, and geographic region as covariates.

Multivariable generalized linear models with a log link and gamma error distribution were used to examine the association between BMI categories, osteoarthritis, and healthcare expenditures. These models used the same specification as described above for the medication‐related regressions.

For all models, adjusted predicted outcomes were generated using the recycled prediction method.^12^ Statistical significance was based upon the model estimates, with Wald chi‐square tests for joint significance of BMI categories; statistical significance tests for trends were also completed by treating BMI as a continuous predictor. A significance threshold of *P* < .05 was used for all analyses. Statistical analyses were conducted using SAS Enterprise Guide 7.1 (SAS Institute Inc, Cary, NC).

### 2.7 Sensitivity analyses

Two sensitivity analyses were undertaken. First, the prevalence of osteoarthritis was recalculated using an expanded definition, which included 715.xx, 716.xx, and 719.xx, as previously examined by Cisternas et al. (2016).^14^ With the use of this expanded definition, the association between BMI categories, osteoarthritis, and healthcare expenditures was also re‐examined. Second, to examine a less restrictive definition of the dichotomous osteoarthritis‐related medication utilization outcomes, these were reanalysed using a threshold of ≥1 prescription fills/medical claims.

### 2.8 Protection of human subjects

The Optum Integrated database consists of de‐identified healthcare records. Use of the database was reviewed by the New England Institutional Review Board (IRB), which determined that it did not constitute human subjects research and was thus exempt from IRB review and registration requirements. Throughout this research project, the study data remained de‐identified and stored on encrypted, password‐protected servers to protect patient confidentiality.

## 3 RESULTS

### 3.1 Patient characteristics

From 2 637 223 patients with ≥1 BMI measure present in the EHR component of the database, a subset of 433 369 had linked insurance claims data and continuous health plan enrolment for 6 months before and 6 months after the index BMI measurement. Applied consecutively, 423 075 patients had no missing data for demographic covariates; 359 807 were age ≥ 18 years as of index BMI; and 256 459 had an index BMI of 25 to 80 kg m^−2^ (final sample size). Of the 256 459 patients, 14.8% (38 050) had osteoarthritis on the basis of the presence of an osteoarthritis *ICD‐9‐CM* diagnosis code recorded in the insurance claims data.

Tables 1 and 2 show patient demographics and clinical characteristics, respectively. Within each BMI category, patients with osteoarthritis were older, had a greater proportion of females, and had a greater proportion of patients with Medicare coverage (Table 1). Patients with osteoarthritis had greater prevalence rates of almost all co‐morbidities contained in the Charlson co‐morbidity index than had patients without osteoarthritis (Table 2). The three most prevalent co‐morbidities were chronic obstructive pulmonary disease (ranging from 12.0% in patients who were overweight and without osteoarthritis to 30.0% in patients with class III obesity and osteoarthritis), diabetes (ranging from 8.5% “uncomplicated”/3.9% “complicated” in patients who were overweight and without osteoarthritis to 27.3% uncomplicated/16.0% complicated in patients with class III obesity and osteoarthritis), and renal disease (ranging from 5.6% in patients who were overweight and without osteoarthritis to 13.4% in patients with class III obesity and osteoarthritis) (Table 2).

**Table 1:** Patient demographics by body mass index (BMI) and osteoarthritis

	BMI Category							
	Overweight		Class I Obese		Class II Obese		Class III Obese	
	Osteoarthritis							
	No	Yes	No	Yes	No	Yes	No	Yes
N	107 188	15 654	64 408	11 582	28 027	5934	18 786	4880
Age, y								
18‐29	10.3%	0.6%	7.8%	0.3%	8.5%	0.4%	9.0%	0.5%
30‐39	13.3%	1.7%	12.4%	1.9%	14.0%	2.2%	16.7%	3.1%
40‐49	16.1%	5.8%	17.5%	6.4%	19.0%	7.8%	21.5%	11.1%
50‐59	20.1%	16.3%	22.5%	19.8%	23.1%	23.1%	23.2%	29.1%
60‐64	8.3%	11.0%	9.1%	12.3%	9.1%	14.4%	9.1%	15.3%
65+	31.9%	64.7%	30.7%	59.4%	26.3%	52.0%	20.6%	40.8%
Female	47.2%	55.4%	47.5%	56.9%	55.0%	63.7%	62.4%	70.9%
Geographic region								
Midwest	43.1%	42.4%	46.3%	46.1%	47.9%	47.0%	48.3%	47.5%
Northeast	12.3%	13.4%	9.7%	10.9%	8.2%	9.0%	7.0%	8.3%
South	30.9%	29.3%	32.7%	30.7%	33.8%	33.8%	35.9%	34.8%
West	13.7%	15.0%	11.3%	12.3%	10.0%	10.1%	8.8%	9.4%
Insurance type								
Commercial	69.9%	39.1%	70.4%	42.2%	72.9%	46.0%	75.9%	50.8%
Medicare	30.1%	60.9%	29.6%	57.8%	27.1%	54.0%	24.1%	49.2%

*Note.* Overweight (BMI 25.0‐29.9 kg m^−2^); class I obese (BMI 30.0‐34.9 kg m^−2^); class II obese (BMI 35.0‐39.9 kg m^−2^); and class III obese (BMI ≥ 40 kg m^−2^).

**Table 2:** Charlson co‐morbidities by body mass index (BMI) and osteoarthritis

	BMI Category							
	Overweight		Class I Obese		Class II Obese		Class III Obese	
	Osteoarthritis							
	No	Yes	No	Yes	No	Yes	No	Yes
N	107 188	15 654	64 408	11 582	28 027	5934	18 786	4880
Cancer	7.5%	12.1%	7.1%	10.4%	6.4%	9.2%	5.5%	8.9%
Cerebrovascular dis.	5.2%	11.0%	4.8%	9.6%	4.2%	9.1%	3.5%	7.2%
COPD	12.0%	19.7%	13.3%	20.7%	15.4%	24.0%	18.5%	30.0%
Dementia	0.8%	2.5%	0.5%	1.7%	0.3%	1.1%	0.3%	0.7%
Diabetes, uncomplicated	3.9%	6.8%	5.6%	9.9%	7.5%	13.3%	9.3%	16.0%
Diabetes, complicated	8.5%	13.0%	13.7%	17.3%	17.8%	22.3%	22.1%	27.3%
Heart failure	4.0%	9.0%	4.6%	9.4%	5.0%	10.3%	6.6%	13.2%
Hemiplegia/paraplegia	0.5%	1.0%	0.5%	0.8%	0.4%	0.7%	0.4%	0.8%
HIV/AIDS	0.3%	0.2%	0.2%	0.1%	0.1%	0.1%	0.1%	0.1%
Liver dis., mild	3.0%	4.7%	4.0%	5.3%	4.6%	6.3%	4.9%	7.0%
Liver dis., mod./sev.	0.2%	0.3%	0.2%	0.3%	0.2%	0.4%	0.3%	0.6%
Myocardial infarction	2.3%	4.0%	2.2%	3.6%	2.1%	3.5%	1.9%	3.6%
Peptic ulcer dis.	0.6%	1.4%	0.6%	1.3%	0.6%	1.0%	0.6%	1.3%
Peripheral vascular dis.	5.4%	12.6%	5.0%	11.6%	4.4%	10.9%	4.4%	10.0%
Renal dis.	5.6%	11.7%	6.1%	12.6%	6.4%	12.5%	6.8%	13.4%
Rheumatologic dis.	1.5%	7.4%	1.6%	6.6%	1.6%	7.2%	1.7%	6.7%

*Note.* Overweight (BMI 25.0‐29.9 kg m^−2^); class I obese (BMI 30.0‐34.9 kg m^−2^); class II obese (BMI 35.0‐39.9 kg m^−2^); and class III obese (BMI ≥ 40 kg m^−2^).

Abbreviations: AIDS, acquired immune deficiency syndrome; COPD, chronic obstructive pulmonary disorder; dis., disease; HIV, human immunodeficiency virus; mod., moderate; sev., severe.

**Figure 1:**
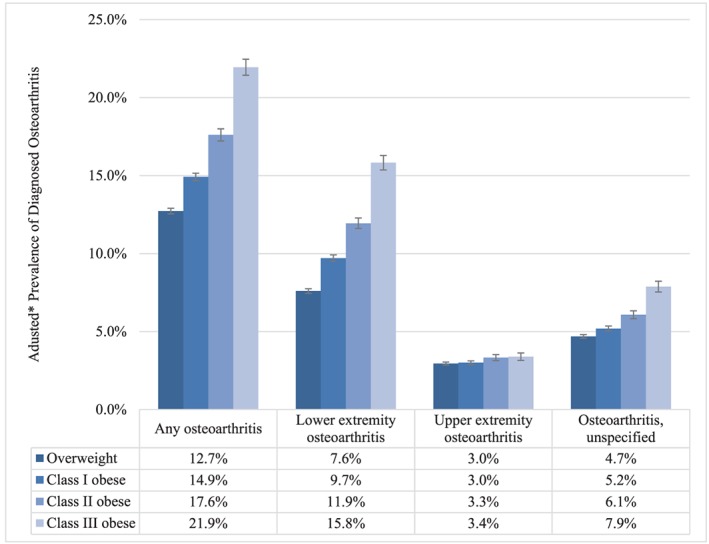
Adjusted prevalence of osteoarthritis by body mass index (BMI) category. Overweight (BMI 25.0‐29.9 kg m^−2^); class I obesity (BMI 30.0‐34.9 kg m^−2^); class II obesity (BMI 35.0‐39.9 kg m^−2^); and class III obesity (BMI ≥ 40 kg m^−2^). *Adjusted for age, sex, and geographic region of residence; error bars represent 95% confidence intervals. Prevalence rates differed significantly across BMI categories/by BMI (*P* < .001 for Wald chi‐square test of joint significance of BMI categories; *P* < .001 for test of trend; except for upper‐extremity osteoarthritis)

**Figure 2:**
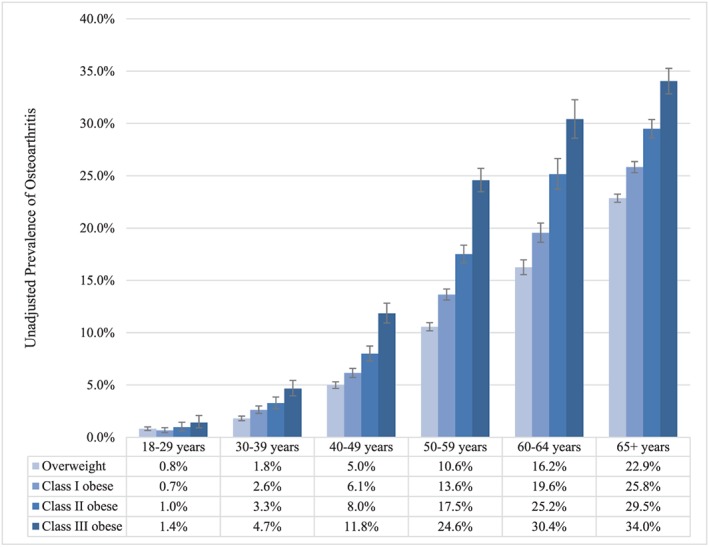
Unadjusted prevalence of osteoarthritis by body mass index (BMI) and age category. Overweight (BMI 25.0‐29.9 kg m^−2^); class I obese (BMI 30.0‐34.9 kg m^−2^); class II obese (BMI 35.0‐39.9 kg m^−2^); and class III obese (BMI ≥ 40 kg m^−2^). Error bars represent 95% confidence intervals

**Figure 3:**
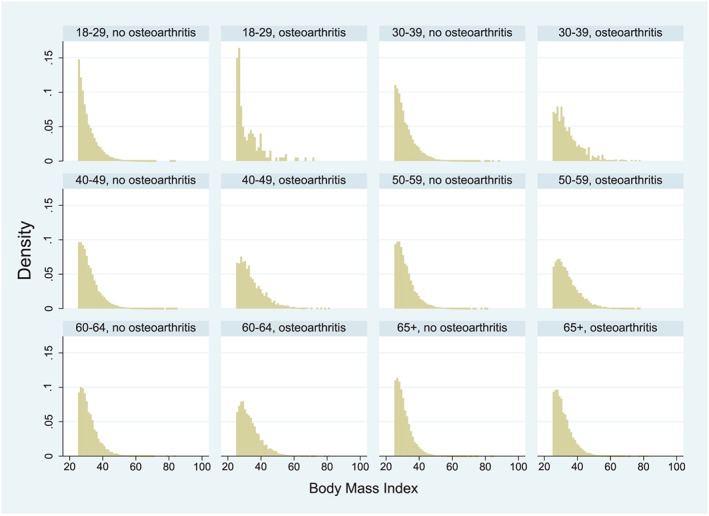
Distribution of body mass index (BMI) by age category and osteoarthritis

Figure 1 shows adjusted prevalence rates of osteoarthritis for each anatomical site, stratified by BMI category. The adjusted prevalence of any osteoarthritis increased monotonically (ie, steadily) within increasing BMI category (*P* < .001), from 12.7% (95% confidence interval, 12.5‐12.9%) among patients who were overweight to 21.9% (95% CI, 21.4‐22.5%) among patients with class III obesity. The increases were driven primarily by the categories of lower extremity and unspecified osteoarthritis; the adjusted prevalence of upper‐extremity osteoarthritis differed by less than half a percentage point across the BMI categories (Figure 1). Figure 2 shows the unadjusted prevalence rate of any osteoarthritis by age category. In all age categories, the unadjusted prevalence of any osteoarthritis increased monotonically within increasing BMI category (Figure 2). Figure 3 shows the distribution of BMI values by age category and presence vs absence of any osteoarthritis. Within each age category, patients with osteoarthritis tended to have distributions of BMI that were more right skewed towards higher BMI.

Table 3 shows adjusted rates of osteoarthritis‐related medication utilization, stratified by BMI category and presence vs absence of any osteoarthritis. Of the examined osteoarthritis‐related medication classes, opioids were the most commonly utilized class, with adjusted prevalence rates of patients with ≥2 prescription fills ranging from 10.5% (95% CI, 10.3‐10.6%) in patients who were overweight and without osteoarthritis to 39.3% (95% CI, 37.9‐40.6%) in patients with class III obesity and osteoarthritis (Table 3). Within each BMI category, osteoarthritis‐related medication utilization rates were higher among patients with osteoarthritis than in patients without (all *P* < .001), and osteoarthritis‐related medication utilization rates tended to increase monotonically with increasing BMI category (most *P* < .001) (Table 3).

**Table 3:** Adjusted osteoarthritis medication use rates, requiring ≥2 prescription fills, by body mass index (BMI) and osteoarthritis

	BMI Category							
	Overweight		Class I Obese		Class II Obese		Class III Obese	
	Osteoarthritis							
	No	Yes	No	Yes	No	Yes	No	Yes
N	107 188	15 654	64 408	11 582	28 027	5934	18 786	4880
Any analgesic^a^	16.4% (16.2‐16.6%)	41.5% (40.7‐42.3%)	18.8% (18.5‐19.0%)	45.1% (44.2‐46.0%)	21.3% (20.8‐21.7%)	48.7% (47.5‐50.0%)	23.7% (23.1‐24.3%)	53.3% (52.0‐54.7%)
Opioids	10.5% (10.3‐10.6%)	28.8% (28.1‐29.5%)	12.2% (11.9‐12.4%)	32.2% (31.4‐33.1%)	13.8% (13.4‐14.2%)	35.0% (33.8‐36.2%)	16.0% (15.5‐16.5%)	39.3% (37.9‐40.6%)
NSAIDs/COX‐2 inhibitors	5.0% (4.9‐5.1%)	16.6% (16.0‐17.2%)	6.2% (6.1‐6.4%)	18.8% (18.1‐19.5%)	7.8% (7.5‐8.1%)	20.7% (19.7‐21.7%)	8.7% (8.3‐9.1%)	22.9% (21.7‐24.0%)
Intra‐articular inj.	0.4% (0.4‐0.5%)	12.1% (11.6‐12.6%)	0.5% (0.4‐0.5%)	14.0% (13.4‐14.6%)	0.5% (0.4‐0.6%)	14.6% (13.7‐15.5%)	0.5% (0.4‐0.6%)	16.1% (15.1‐17.1%)
Muscle relaxants	2.7% (2.6‐2.8%)	6.8% (6.4‐7.3%)	3.3% (3.1‐3.4%)	7.7% (7.2‐8.2%)	3.6% (3.4‐3.9%)	7.9% (7.2‐8.5%)	3.8% (3.6‐4.1%)	8.8% (8.1‐9.6%)
Antidepressants	14.7% (14.5‐15.0%)	19.5% (18.9‐20.1%)	16.2% (15.9‐16.4%)	21.8% (21.0‐22.5%)	17.7% (17.3‐18.1%)	23.5% (22.5‐24.6%)	18.7% (18.1‐19.2%)	24.8% (23.7‐26.0%)
Anticonvulsants	4.9% (4.8‐5.1%)	9.2% (8.8‐9.6%)	5.9% (5.7‐6.0%)	10.6% (10.1‐11.2%)	6.8% (6.5‐7.1%)	12.5% (11.7‐13.2%)	8.0% (7.6‐8.4%)	14.6% (13.7‐15.5%)
Anx./sed./hyp.	9.5% (9.3‐9.7%)	14.7% (14.2‐15.3%)	9.7% (9.4‐9.9%)	13.9% (13.3‐14.6%)	9.6% (9.3‐10.0%)	14.8% (13.9‐15.7%)	9.7% (9.3‐10.1%)	14.4% (13.4‐15.3%)

*Note.* Overweight (BMI 25.0‐29.9 kg m^−2^); class I obese (BMI 30.0‐34.9 kg m^−2^); class II obese (BMI 35.0‐39.9 kg m^−2^); and class III obese (BMI ≥ 40 kg m^−2^). Medication utilization rates differed significantly between patients with vs without osteoarthritis (*P* < .001), and across BMI categories/by BMI (*P* < .001 for Wald chi‐square test of joint significance of BMI categories; *P* < .001 for test of trend; except for intra‐articular injections [*P* = .0816 for test of trend]; anxiolytics, sedatives, hypnotics [*P* = .3745 for test of trend]).

Abbreviations: Anx./sed./hyp., anxiolytics, sedatives, hypnotics; inj., injections; NSAIDs, nonsteroidal anti‐inflammatory drugs.

a Prescription fills for any of the following categories: opioids, NSAIDs/COX‐2 inhibitors, acetaminophen, and salicylates.

**Figure 4:**
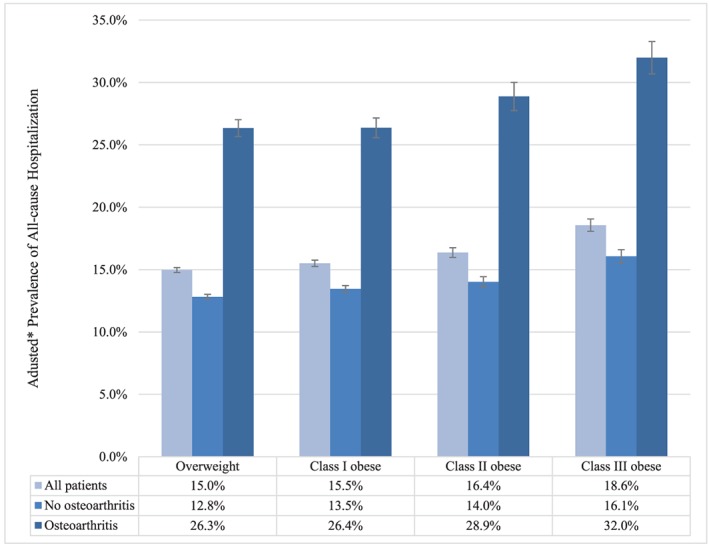
Adjusted 1‐y prevalence of all‐cause hospitalization by body mass index (BMI) category and osteoarthritis. Overweight (BMI 25.0‐29.9 kg m^−2^); class I obesity (BMI 30.0‐34.9 kg m^−2^); class II obesity (BMI 35.0‐39.9 kg m^−2^); and class III obesity (BMI ≥ 40 kg m^−2^). *Adjusted for age, sex, and geographic region of residence; error bars represent 95% confidence intervals. The prevalence of all‐cause hospitalization differed significantly between patients with vs without osteoarthritis (*P* < .001) and across BMI categories/by BMI (*P* < .001 for Wald chi‐square test of joint significance of BMI categories; *P* < .001 for test of trend)

Figure 4 shows adjusted prevalence of all‐cause hospitalization over the 1‐year evaluation period, stratified by BMI category and presence vs absence of any osteoarthritis. The adjusted prevalence of all‐cause hospitalization ranged from 12.8% (95% CI, 12.6‐13.0%) in patients who were overweight and without osteoarthritis to 32.0% (95% CI, 30.7‐33.3%) among patients with class III obesity and osteoarthritis (Figure 4). The adjusted prevalence of all‐cause hospitalization increased monotonically with increasing BMI category (*P* < .001), and within each BMI category, it was approximately two times higher among patients with osteoarthritis than among those without (*P* < .001) (Figure 4). To explore the extent to which osteoarthritis was directly responsible for the greater utilization of inpatient services, the proportion of patients with osteoarthritis who were hospitalized for osteoarthritis and/or lower‐extremity orthopaedic surgery was evaluated. Table 4 shows unadjusted rates of hospitalization with a primary diagnosis of osteoarthritis and/or a lower‐extremity orthopaedic surgery DRG (primary or revision). Across the BMI categories, approximately one third of all‐cause hospitalizations had a primary diagnosis of osteoarthritis (3290/11 318 hospitalizations), and 80.9% to 87.0% of hospitalizations with a primary diagnosis of osteoarthritis were for a lower‐extremity orthopaedic surgery DRG (Table 4). The unadjusted rates of hospitalizations with primary diagnoses and/or lower‐extremity orthopaedic surgery DRG increased from the overweight category to the obese class II category and were approximately equal for the obese class II and obese class III categories (Table 4).

**Table 4:** Unadjusted prevalence of osteoarthritis‐related or orthopaedic surgery‐related hospitalization by body mass index category, among patients with osteoarthritis

	BMI Category			
	Overweight	Class I Obese	Class II Obese	Class III Obese
N	15 654	11 582	5934	4880
Hospitalization type				
All‐cause	29.3% (28.6‐30.0%)	28.6% (27.8‐29.5%)	30.7% (29.5‐31.9%)	32.7% (31.4‐34.1%)
Primary diagnosis of osteoarthritis	7.4% (7.0‐7.8%)	9.1% (8.6‐9.6%)	10.2% (9.4‐11.0%)	9.8% (8.9‐10.6%)
Lower‐extremity orthopaedic surgery DRG^a^	7.2% (6.8‐7.6%)	8.7% (8.2‐9.2%)	9.5% (8.7‐10.2%)	9.5% (8.7‐10.3%)
Primary diagnosis of osteoarthritis and lower‐extremity orthopaedic surgery DRG^a^	6.0% (5.6‐6.4%)	7.9% (7.4‐8.4%)	8.4% (7.7‐9.2%)	8.5% (7.7‐9.3%)

*Note.* Overweight (BMI 25.0‐29.9 kg m^−2^); class I obese (BMI 30.0‐34.9 kg m^−2^); class II obese (BMI 35.0‐39.9 kg m^−2^); and class III obese (BMI ≥ 40 kg m^−2^).

Abbreviation. DRG, diagnosis‐related group.

a Primary or revision.

**Figure 5:**
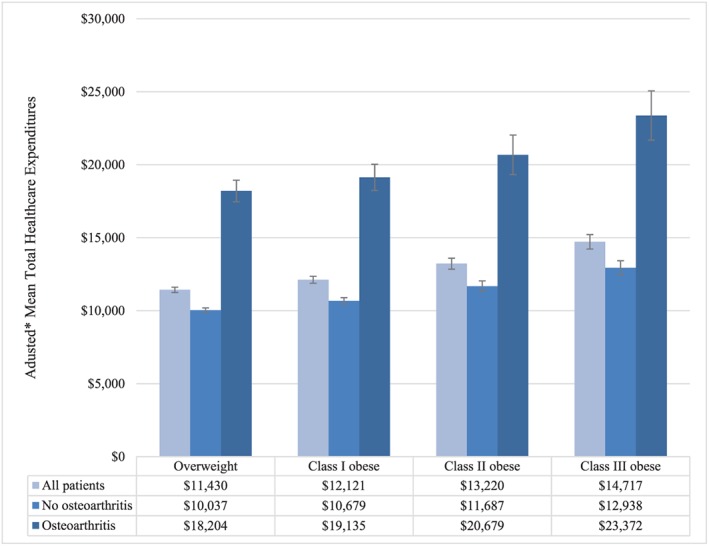
Adjusted mean total healthcare expenditures by body mass index (BMI) category and osteoarthritis. Overweight (BMI 25.0‐29.9 kg m^−2^); class I obesity (BMI 30.0‐34.9 kg m^−2^); class II obesity (BMI 35.0‐39.9 kg m^−2^); and class III obesity (BMI ≥ 40 kg m^−2^). *Adjusted for age, sex, and geographic region of residence; error bars represent 95% confidence intervals. Total healthcare expenditures differed significantly between patients with vs without osteoarthritis (*P* < .001), and across BMI categories/by BMI (*P* < .001 for Wald chi‐square test of joint significance of BMI categories; *P* < .001 for test of trend)

Figure 5 shows adjusted healthcare expenditures, stratified by BMI category and presence vs absence of any osteoarthritis. Adjusted healthcare expenditures ranged from $10 037 (95% CI, $9878‐$10 196) among patients who were overweight and without osteoarthritis to $23 372 ($21 685‐$25 059) among patients with class III obesity and osteoarthritis (Figure 5). Adjusted healthcare expenditures increased monotonically with increasing BMI category (*P* < .001), and within each BMI category, they were nearly 80% higher among patients with osteoarthritis than among those without (*P* < .001) (Figure 5). Table 5 shows unadjusted healthcare expenditures by category. Medical costs, including inpatient and outpatient costs, accounted for the majority of total costs (Table 5). Osteoarthritis‐related medication costs accounted for a relatively small proportion of total costs, ranging from 2.0% in patients who were overweight and without osteoarthritis to 3.5% in patients with class III obesity and osteoarthritis (Table 5).

**Table 5:** Unadjusted mean healthcare expenditures by category, body mass index (BMI), and osteoarthritis

	BMI Category							
	Overweight		Class I Obese		Class II Obese		Class III Obese	
	Osteoarthritis							
	No	Yes	No	Yes	No	Yes	No	Yes
N	107 188	15 654	64 408	11 582	28 027	5934	18 786	4880
Total, mean	$9919	$20 778	$10 484	$21 398	$11 113	$22 812	$12 136	$25 531
Standard deviation	$27 764	$35 636	$27 778	$38 522	$29 119	$38 213	$27 492	$46 337
Median	$2980	$9421	$3365	$10 003	$3730	$11 002	$4161	$12 659
Medical, mean	$7930	$17 462	$8294	$17 838	$8716	$18 823	$9307	$20 786
Standard deviation	$23 829	$31 254	$24 084	$34 433	$25 457	$33 250	$23 070	$42 902
Median	$2005	$7116	$2143	$7408	$2307	$8034	$2489	$8797
Pharmacy,^a^ mean	$1989	$3316	$2190	$3560	$2397	$3990	$2829	$4745
Standard deviation	$10 331	$11 104	$9086	$10 970	$9880	$11 605	$10 665	$11 194
Median	$264	$850	$342	$1009	$408	$1249	$496	$1535
Osteoarthritis related, mean	$196	$545	$235	$641	$269	$769	$309	$905
Standard deviation	$955	$1510	$1101	$1698	$1090	$1856	$1078	$1965
Median	$0	$100	$2	$137	$6	$181	$10	$229

*Note.* Overweight (BMI 25.0‐29.9 kg m^−2^); class I obese (BMI 30.0‐34.9 kg m^−2^); class II obese (BMI 35.0‐39.9 kg m^−2^); and class III obese (BMI ≥ 40 kg m^−2^).

a Includes osteoarthritis‐related medications and other medications; osteoarthritis‐related medications includes opioid analgesics, nonsteroidal anti‐inflammatory drugs (including COX‐2 inhibitors), acetaminophen/salicylates, intra‐articular injections, muscle relaxants, antidepressants, anticonvulsants, and anxiolytic/sedative/hypnotic medications.

### 3.2 Sensitivity analyses

**Figure 6:**
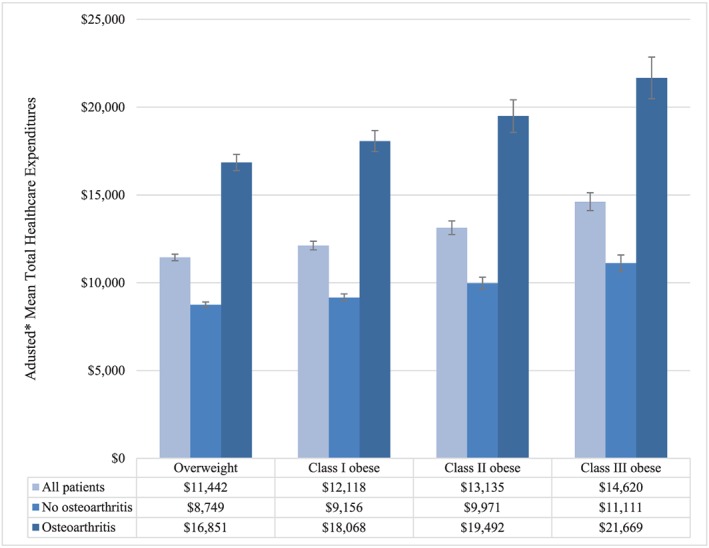
Adjusted mean total healthcare expenditures by body mass index (BMI) category and osteoarthritis (expanded definition). Overweight (BMI 25.0‐29.9 kg m^−2^); class I obese (BMI 30.0‐34.9 kg m^−2^); class II obese (BMI 35.0‐39.9 kg m^−2^); and class III obese (BMI ≥ 40 kg m^−2^). *Adjusted for age, sex, and geographic region of residence; error bars represent 95% confidence intervals. Total healthcare expenditures differed significantly between patients with vs without osteoarthritis (*P* < .001), and across BMI categories/by BMI (*P* < .001 for Wald chi‐square test of joint significance of BMI categories; *P* < .001 for test of trend)

Table 6 shows unadjusted and adjusted prevalence rates of the expanded definition of any osteoarthritis, stratified by BMI category. Compared with the primary analyses' case definition of osteoarthritis, the expanded definition yielded prevalence rates that were approximately two times higher (Table 6). Figure 6 shows adjusted healthcare expenditures, stratified by BMI category and presence vs absence of the expanded definition of any osteoarthritis. Despite yielding substantially different prevalence rates than the case definition of osteoarthritis used in the primary analyses, the relationships between costs and the expanded definition osteoarthritis were very similar to those seen in the primary analyses (Figure 6).

**Table 6:** Unadjusted and adjusted prevalence of diagnosed osteoarthritis by body mass index category, expanded definition

	BMI Category			
	Overweight	Class I Obese	Class II Obese	Class III Obese
N	122 842	75 990	33 961	23 666
Unadjusted prevalence rate	28.4% (28.2‐28.7%)	31.2% (30.9‐31.6%)	34.1% (33.6‐34.6%)	37.3% (36.7‐37.9%)
Adjusted prevalence rate**	28.5% (28.2‐28.7%)	30.9% (30.6‐31.3%)	34.2% (33.7‐34.6%)	38.0% (37.4‐38.6%)

*Note.* Expanded definition includes *International Classification of Diseases, Ninth Revision, Clinical Modification* diagnosis codes of 715.xx, 716.xx, and 719.xx; prevalence rates are adjusted for age, sex, and geographic region of residence. Overweight (BMI 25.0‐29.9 kg m^−2^); class I obese (BMI 30.0‐34.9 kg m^−2^); class II obese (BMI 35.0‐39.9 kg m^−2^); and class III obese (BMI ≥ 40 kg m^−2^).

** *P* < .001 for Wald chi‐square test of joint significance of BMI categories; *P* < .001 for test of trend.

Table 7 shows the results of the sensitivity analysis in which the dichotomous osteoarthritis‐related medication utilization outcomes were reanalysed using a less restrictive threshold of ≥1 prescription fill/medical claim. These results were consistent with the primary analyses; however, whereas the overall osteoarthritis‐related medication utilization rates were higher, the relative rates of medication utilization among patients with vs without osteoarthritis were slightly lower (on average, 25% lower across the categories) (Table 7).

**Table 7:** Adjusted osteoarthritis medication use rates, requiring ≥1 prescription fills, by body mass index (BMI) and osteoarthritis

	BMI Category							
	Overweight		Class I Obese		Class II Obese		Class III Obese	
	Osteoarthritis							
	No	Yes	No	Yes	No	Yes	No	Yes
N	107 188	15 654	64 408	11 582	28 027	5934	18 786	4880
Any analgesic^a^	31.1% (30.8‐31.3%)	57.6% (56.8‐58.4%)	33.8% (33.4‐34.1%)	60.5% (59.7‐61.4%)	36.6% (36.1‐37.2%)	63.6% (62.4‐64.8%)	38.9% (38.3‐39.6%)	66.5% (65.2‐67.8%)
Opioids	23.1% (22.8‐23.3%)	45.8% (45.0‐46.6%)	25.5% (25.2‐25.8%)	48.8% (47.9‐49.7%)	27.7% (27.2‐28.3%)	53.1% (51.8‐54.3%)	30.1% (29.4‐30.7%)	54.7% (53.3‐56.1%)
NSAIDs/COX‐2 inhibitors	13.4% (13.2‐13.6%)	31.7% (30.9‐32.4%)	15.0% (14.7‐15.3%)	33.5% (32.6‐34.4%)	16.9% (16.5‐17.3%)	34.2% (33.0‐35.4%)	17.7% (17.2‐18.2%)	36.0% (34.7‐37.3%)
Intra‐articular inj.	2.3% (2.2‐2.4%)	28.7% (27.9‐29.4%)	2.5% (2.4‐2.6%)	31.4% (30.6‐32.3%)	2.5% (2.4‐2.7%)	33.2% (32.0‐34.3%)	2.5% (2.2‐2.7%)	35.7% (34.3‐37.0%)
Muscle relaxants	7.1% (7.0‐7.3%)	13.5% (12.9‐14.1%)	7.9% (7.7‐8.1%)	14.8% (14.1‐15.6%)	8.6% (8.3‐8.9%)	15.7% (14.7‐16.7%)	8.8% (8.4‐9.1%)	16.4% (15.3‐17.4%)
Antidepressants	16.9% (16.7‐17.2%)	22.4% (21.7‐23.1%)	18.3% (18.0‐18.6%)	24.7% (23.9‐25.5%)	20.0% (19.5‐20.5%)	26.0% (24.9‐27.1%)	21.0% (20.4‐21.5%)	27.5% (26.3‐28.7%)
Anticonvulsants	6.4% (6.3‐6.6%)	12.0% (11.5‐12.5%)	7.5% (7.3‐7.7%)	13.4% (12.8‐14.0%)	8.5% (8.2‐8.8%)	15.5% (14.7‐16.4%)	9.8% (9.4‐10.2%)	17.2% (16.2‐18.2%)
Anx./sed./hyp.	14.4% (14.2‐14.6%)	21.3% (20.7‐22.0%)	14.4% (14.2‐14.7%)	20.9% (20.2‐21.7%)	14.4% (14.0‐14.8%)	21.7% (20.7‐22.7%)	14.5% (14.0‐14.9%)	21.4% (20.3‐22.5%)

*Note.* Overweight (BMI 25.0‐29.9 kg m^−2^); class I obese (BMI 30.0‐34.9 kg m^−2^); class II obese (BMI 35.0‐39.9 kg m^−2^); and class III obese (BMI ≥ 40 kg m^−2^). Medication utilization rates differed significantly between patients with vs without osteoarthritis (*P* < .001), and across BMI categories/by BMI (*P* < .001 for Wald chi‐square test of joint significance of BMI categories; *P* < .001 for test of trend; except for: intra‐articular injections [*P* = .1125 for test of trend]; anxiolytics, sedatives, hypnotics [*P* = .6617 for test of trend]).

Abbreviations: Anx./sed./hyp., anxiolytics, sedatives, hypnotics; inj., injections; NSAID, nonsteroidal anti‐inflammatory drugs.

a Any prescription fill for any of the following categories: opioids, NSAIDs, COX‐2 inhibitors, acetaminophen, and salicylates.

## 4 DISCUSSION

This study sought to quantify the association of osteoarthritis and BMI with healthcare utilization and expenditures in adults aged ≥18 years who were overweight or obese. Through an analysis of a uniquely rich dataset that provided patient information on both BMI and healthcare utilization and expenditures, three key findings arise that are of relevance to the management of osteoarthritis and obesity: (a) increasing BMI is associated with an increasing prevalence of osteoarthritis; (b) even when adjusting for BMI, osteoarthritis has a strong independent association with substantially increased healthcare utilization and expenditures; and (c) among patients with osteoarthritis, increasing BMI is associated with increasing healthcare utilization and expenditures. These relationships are intuitive; however, to our knowledge, this study is the first to empirically quantify the magnitude of the relationships. Although this study did not directly test the impact of weight loss on prevention or outcomes of osteoarthritis, this study contributes novel information that can be used to understand what potential opportunity exists, if any, to derive benefit from interventions to reduce obesity in the context of osteoarthritis prevention and management.

Increasing BMI was associated with an increasing prevalence of osteoarthritis—we found that patients with class III obesity had an adjusted prevalence rate of osteoarthritis that was nearly two times higher than that of patients who were overweight (21.9% vs 12.7%, respectively); this trend was also evident when stratified by age category. This was driven primarily by lower‐extremity osteoarthritis, which includes osteoarthritis of the knee and hip and which was the most prevalent type of osteoarthritis. Of the lower‐extremity diagnoses recorded during the evaluation period, 66.2% were for the lower leg (knee), 21.2% were for the pelvic region and thigh (hip), and 8.6% were for the ankle and foot. Although upper‐extremity osteoarthritis increased only slightly with increasing BMI, the adjusted prevalence rate of osteoarthritis of “unspecified” sites was also 68% higher among patients with class III obesity (7.9%) as compared with patients who were overweight (4.7%). The pronounced findings in the lower‐extremity regions are consistent with the mechanical relationship between obesity and excessive joint loading.^5^

Even when adjusting for BMI, osteoarthritis has a strong independent association with substantially increased healthcare utilization and expenditures—previous analyses of the economic burden of osteoarthritis have uniformly reported greater healthcare expenditures among patients with osteoarthritis as compared with those without it.^4^, ^5^, ^6^ Because these previous analyses were unable to account for the influence of BMI, it was possible that some of the observed expenditure differences could have been driven partially by imbalances in obesity across those with vs without osteoarthritis. Specifically, because increasing BMI is associated with both a greater prevalence of osteoarthritis and higher healthcare utilization and expenditures, prior estimates may have overestimated the burden of osteoarthritis. In a previous analysis that used a methodology closely resembling that of the present study, but not accounting for BMI, Le et al^4^ reported that patients with osteoarthritis had healthcare expenditures that were double those of patients without osteoarthritis. In the present analysis, a slightly lower magnitude of association (~80% higher) was seen between the presence of osteoarthritis and healthcare expenditures, and this relationship held across all examined BMI categories. Thus, the present study empirically confirms the strong independent association between osteoarthritis and substantially increased healthcare utilization and expenditures.

Among patients with osteoarthritis, increasing BMI is associated with increasing healthcare utilization and expenditures. The increased expenditures across BMI categories were driven by greater general and osteoarthritis‐related pharmacy utilization, greater all‐cause hospitalization rates, and greater osteoarthritis/orthopaedic surgery‐related hospitalization rates. Use of analgesics and other osteoarthritis‐related medications, including opioid and nonopioid medications, was higher among patients with osteoarthritis and increased with increasing BMI among both those with and those without osteoarthritis. These findings are consistent with the concept that increasing BMI, particularly to levels of class III obesity, is associated with increased osteoarthritis disease severity/pain (more than 35% of patients with class II and III obesity were taking opioids) and increased need for surgical treatment of knee osteoarthritis through total knee arthroplasty (TKA).^15^, ^16^Because the study's data source did not have information on the reason medications were prescribed, further research is warranted to understand specific conditions for which the medications, such as opioids, were intended.

Knee osteoarthritis is the primary reason for knee arthroplasty and imposes a substantial burden on the US healthcare system. For individuals who are candidates for knee arthroplasty, weight loss may be particularly important, as several studies have associated obesity (and particularly class III obesity) with an increased risk of complications, such as infection and readmissions, and increased hospital costs in patients undergoing primary or revision TKA.^17^, ^18^ Consequently, patients with obesity may be advised to lose weight before undergoing knee arthroplasty. To adequate levels, weight loss before knee arthroplasty may not only have the potential to improve perioperative outcomes but could potentially delay or avoid the need for surgical intervention.

Bariatric surgery is the most effective and durable means of weight loss for class II/III obesity.^14^, ^19^ However, there is a paucity of evidence on the effects of any weight loss technique on perioperative and long‐term outcomes of TKA. Future studies are needed understand the extent to which interventions—including weight loss or prevention of obesity progression—would have a meaningful impact on the healthcare burden associated with osteoarthritis. The findings from this study suggest that great benefits may result from such interventions, both through primary prevention of weight‐related osteoarthritis and through secondary weight management among patients with osteoarthritis.

This study was subject to limitations. First, the Optum Integrated database is a nonprobability sample, and these data may not be generalizable to the entire US population. Furthermore, only individuals who had contact with a healthcare provider, a necessary condition to have a BMI value recorded during the study window, were included for study, and therefore, these data correspond to a selected population.

Second, osteoarthritis was defined using ≥1 medical claim with an *ICD‐9‐CM* diagnosis code for osteoarthritis and allied conditions (715.xx) recorded in any diagnosis position. Previous studies have suggested that this case definition may be very restrictive, with relatively low sensitivity (34.6%) but high specificity (97.5%).^14^ However, our sensitivity analyses yielded findings that were generally consistent with the primary analyses when examining the relative healthcare expenditures between patients with vs without an expanded definition of osteoarthritis. Thus, the public health burden associated with osteoarthritis may be even larger than previously estimated using more restrictive case definitions.

Third, the database did not have information on disability, functioning, pain, or workplace absenteeism and presenteeism, among other potentially interesting patient‐reported measures. These unmeasured aspects of the clinical and economic burden of osteoarthritis contribute substantially to the individual, caregiver, and societal burden of osteoarthritis,^13^ and their variation by BMI among patients with osteoarthritis remains as an important topic for future research. Additionally, the database did not have information on race/ethnicity, socio‐economic status, or lifestyle habits such as alcohol consumption, smoking history, or diet and exercise habits, which could potentially confound the relationships observed for the present study.

Finally, because of the cross‐sectional nature of the present study, longitudinal changes in BMI could not be associated with the other outcomes of interest, and duration of obesity as a measure of cumulative risk was unavailable. However, based on a large body of evidence, guidelines recommend that individuals with osteoarthritis lose weight and participate in exercise.^20^

## 5 CONCLUSION

In summary, increasing BMI was associated with an increasing prevalence of osteoarthritis, and increasing BMI and presence of osteoarthritis were both independently associated with increasing healthcare utilization and expenditures. These findings quantify a large opportunity that exists to reduce the healthcare burden associated with osteoarthritis, potentially through primary prevention of weight‐related osteoarthritis, and through secondary weight management among patients with osteoarthritis.

## CONFLICT OF INTEREST

Stephen Johnston is an employee and stockholder of Johnson & Johnson. Eric Ammann is an employee of Johnson & Johnson. Robin Scamuffa was an employee and stockholder of Ethicon, a Johnson & Johnson company, at the time of this research. Jonathan Samuels has no financial interests to disclose. Andrew Stokes has received research funding from Ethicon, a Johnson & Johnson company, for an unrelated study. Elliott Fegelman was an employee and stockholder of Ethicon, a Johnson & Johnson company, at the time of this research. Carine Hsiao was an employee and stockholder of Ethicon, a Johnson & Johnson company, at the time of this research.

## AUTHOR CONTRIBUTIONS

S.J. and E.A. contributed to study design, data collection, data analysis, data interpretation, literature search, generation of figures, and writing of the manuscript. R.S., E.F., A.S., and C.H. contributed to study design, data interpretation, and writing of the manuscript. J.S. contributed to data interpretation and writing of the manuscript.
